# Explainable AI for Digital Health: Predicting Depression Risk in Older Adults Living Alone Using Machine Learning

**DOI:** 10.3390/healthcare14183088

**Published:** 2026-09-19

**Authors:** Dong-Geon Lee, Bum-Jeun Seo, Mi-Joon Lee, Ye-Eun Lee, Eun-A Kim

**Affiliations:** 1 Metabolism-Dementia Research Institute, Yonsei University College of Medicine, Seoul 03722, Republic of Korea; ldg2818@gmail.com; 2 Department of Medical Information, Kongju National University, Gongju-si 32588, Republic of Korea; yeaeun515@naver.com (Y.-E.L.); eua5306@naver.com (E.-A.K.)

**Keywords:** older adults living alone, depression, pain, machine learning, explainable AI, digital health

## Abstract

**Background**: This study aimed to evaluate the performance of machine learning models in predicting depression risk among older adults living alone and to identify the features contributing to those predictions using explainable artificial intelligence (XAI). **Methods**: We analysed 2022 nationwide survey data in Korea. A total of 1007 older adults remained after excluding respondents who lived in multi-person households, were aged < 65 years, or had physician-diagnosed dementia. Depression risk was defined using the CES-D-10 (cutoff ≥ 10). After removing features with high multicollinearity, logistic LASSO selected 23 predictors. Six algorithms were fitted using the training set, with hyperparameter tuning performed by 5-fold cross-validation where applicable, and evaluated in a held-out test set following a 70/30 split. SMOTE was applied only to the training data. Performance was summarised using AUC, sensitivity, specificity and the F1 score with bootstrap 95% confidence intervals, and stability was assessed by repeated stratified cross-validation. SHAP values provided explainability. **Results**: LightGBM achieved an AUC of 0.802 (95% CI 0.747–0.852), followed by Random Forest (0.794) and Logistic Regression (0.779). These differences were small relative to the uncertainty of the estimates. SHAP analysis identified oral health-related quality of life, satisfaction with relationships with children, frequency of social contact, overall life satisfaction, satisfaction with health status, and age as the most influential features. IADL limitations, diabetes, hypertension, and perceived social class contributed to predictions with smaller effects. **Conclusions**: An explainable LightGBM model achieved an AUC of 0.802 for depression risk among older adults living alone and identified psychosocial and health-related features, particularly oral health and social connectedness, that may help inform future screening strategies.

## 1. Introduction

In Korea, adults aged 65 years or older comprise 20.3% of the total population, and this proportion is expected to continue rising annually [1]. Notably, in 2023, among households headed by individuals aged 65 and older, 2.138 million households—representing 37.8%—consisted of a single person living alone, a figure that has been steadily increasing since 32.9% in 2015 [2]. As time passes, older couples are more likely to transition into solitary living due to spousal death. Given the ongoing transformation of the family structure in modern society, the increase in the population of older adults living alone appears inevitable [3]. These individuals tend to have limited contact not only with family members but also with others in society, and they are often burdened with various chronic illnesses, leading to social isolation and challenges in daily functioning [4]. Compared to their non-single-living counterparts, older adults living alone face more vulnerable environments and are more likely to experience not only financial and physical difficulties but also significant emotional distress, necessitating increased attention to their risk of depression [5].

Depression in older adults is frequently triggered by exposure to negative conditions resulting from declining physical, social, and economic functioning. If depressive symptoms persist, they can lead to severe consequences such as suicidal ideation, thereby adversely affecting overall life satisfaction [6]. Previous studies have demonstrated that individuals living alone exhibit higher levels of depression than those in other household types, regardless of gender [7]. Among older adults living alone, depression may manifest not only in psychological symptoms such as frustration, loneliness, and loss of motivation, but also in physical symptoms including sleep disorders, weight loss, loss of appetite, and cognitive decline. In severe cases, these may result in self-neglect and suicidal thoughts or attempts [8].

Earlier studies examining the factors influencing depression among older adults living alone have largely employed traditional statistical techniques, focusing on linear associations and the independent effects of individual variables. However, such approaches are limited in capturing interactions among variables or identifying complex patterns [9,10]. Consequently, recent research has increasingly employed big data analytics and machine learning (ML) techniques to predict depression in older adults [11,12].

Despite their predictive strength, ML models often face the “black box” problem, wherein the interpretability of predictions is limited [13]. To address this, explainable artificial intelligence (XAI) techniques can be employed to provide interpretability at the level of individual predictions, thereby enhancing the transparency and trustworthiness of model outputs [14,15]. Moreover, XAI methods allow visualization of the decision-making process by showing how specific features influence model predictions, which can contribute to improved credibility [16,17].

Current research has investigated a range of demographic, economic, health-related, and quality-of-life factors that influence depression in older adults living alone. Demographically, older men, those with lower education levels (middle school or less), recipients of basic livelihood support, and individuals who do not utilize senior centers or welfare services have been found to be at higher risk for depression [18,19]. The absence of religious participation also contributes to increased depressive symptoms in this population [20]. Moreover, those who are bereaved, divorced, or never married, as well as those who have infrequent contact with close acquaintances, tend to exhibit elevated levels of depression [21]. Medical aid recipients are also more likely to experience depressive symptoms compared to those covered by national health insurance [22].

Economically inactive older adults report higher levels of depression compared to those who are economically active, potentially due to the financial decline associated with retirement [23]. Higher living expenses were associated with greater life satisfaction and lower depression, while financial support from children significantly alleviated depressive symptoms [5,24]. Furthermore, older adults with greater personal assets reported lower depression levels and better overall mental health and life satisfaction [25].

In terms of health-related factors, individuals with a BMI below 25, those who experience high levels of stress, or those with activity limitations are at greater risk for depression [26,27]. The risk also increases with physical impairments, poorer subjective health, and a higher number of chronic conditions [28,29]. More frequent and prolonged pain exacerbates depressive symptoms, whereas regular physical activity has been shown to alleviate them [30,31]. More than one-third of older adults diagnosed with mild cognitive impairment also experience depression, indicating a strong link between cognitive decline and depressive symptoms [32].

Regarding quality of life, higher depression levels are associated with lower life satisfaction. Poor oral health is also linked to reduced life satisfaction and increased depression, suggesting a strong interconnection between these factors [33]. A positive relationship with children has been shown to significantly impact subjective class awareness, depression, and life satisfaction, particularly among older women living alone [34,35].

This study seeks to develop a machine learning-based predictive model for identifying depression risk among older adults aged 65 and older living alone, using the 9th wave of the KLoSA, specifically its structured and light versions. The specific objectives are as follows:

1. To identify general characteristics associated with depression risk in older adults living alone.
2. To develop an effective machine learning model for predicting depression risk using various ML algorithms.
3. To explore key predictors influencing depression risk using SHAP, an explainable AI technique.

## 2. Materials and Methods

### 2.1. Study Design and Subjects

This study utilized data from the 9th wave (2022) of the KLoSA, provided by the Korea Employment Information Service (KEIS) [36]. The KLoSA was established to collect foundational data for the formulation of effective socioeconomic policies by measuring various attributes of the aging population, including their social, economic, psychological, demographic, and health-related conditions. The baseline KLoSA sample was constructed in 2006 with 10,254 individuals aged 45 and older (born before 1961) residing in South Korea, excluding Jeju Island. In 2014, an additional 920 individuals born between 1962 and 1963 were included. Since the release of the 6th wave in 2016, the dataset has been made available in three formats: raw data, structured (reshaped) data, and a light version.

**Figure 1:**
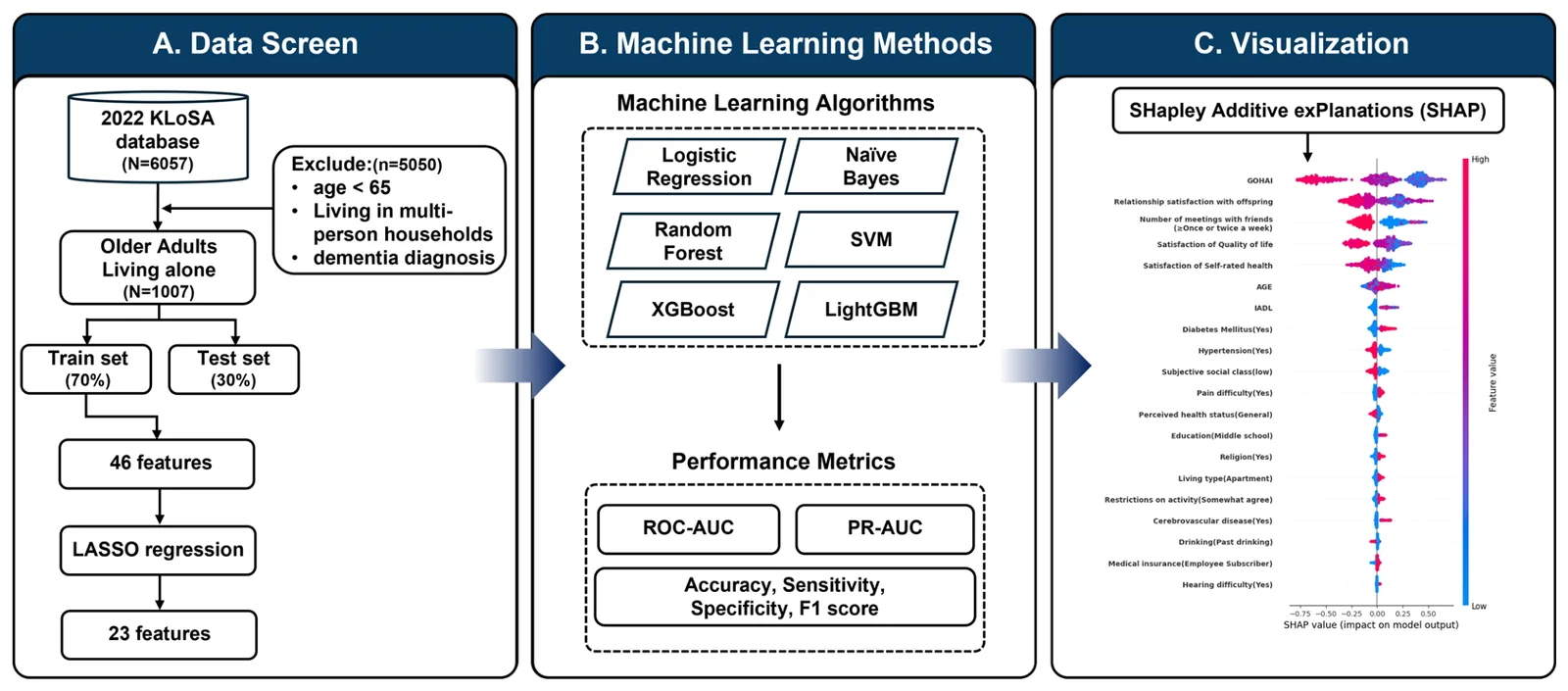
The overall flowchart of the study. (**A**) Data Screen: selection of the analytic sample from the 2022 KLoSA database (N = 6057) to older adults living alone (N = 1007), the training–test split, and feature reduction by LASSO regression (Supplementary Figures S1 and S2); (**B**) Machine Learning Methods: the six algorithms trained and the measures used to evaluate them; (**C**) Visualization: SHAP applied to the final model.

For this study, both the structured data and light version from the 9th wave were merged to incorporate a broader set of variables. Of the 6057 total respondents, 5050 were excluded based on the following criteria: living in multi-person households, being under the age of 65, or having physician-diagnosed dementia that precluded reliable survey responses. The final analytical sample comprised 1007 older adults living alone. The overall data processing and analysis flow is illustrated in Figure 1.

### 2.2. Variables

#### 2.2.1. Dependent Variable

The dependent variable in this study was the presence of depression risk. Depression was measured using the 10-item Korean version of the Center for Epidemiological Studies Depression Scale (CES-D), which was adapted from the original 20-item scale developed in the United States for older adults and individuals with chronic illness. Each item was rated on a 4-point Likert scale: 0 (rarely, ≤1 day), 1 (sometimes, 1–2 days), 2 (frequently, 3–4 days), and 3 (always, ≥5 days). A higher total score indicated greater risk of depression.

The Cronbach alpha coefficient of the CES-D-10 was 0.853 in a previous KLoSA-based study [37] and 0.85 in the present study, indicating good internal consistency. The total score ranged from 0 to 30. After reverse coding, a cutoff score of 10 was used to classify depression risk, in accordance with established guidelines [38,39]. Individuals scoring below 10 were categorized as “no depression risk” (0), and those scoring 10 or above were classified as “at risk of depression” (1).

#### 2.2.2. Independent Variables

Demographic factors included gender, age, education level, marital status, religion, frequency of contact with close acquaintances, type of residence, residential region, and type of medical coverage (National Health Insurance or Medical Aid). Family-related variables included the number of surviving siblings and the total amount of financial support received from family members.

Health status variables included Activities of Daily Living (ADL), Instrumental Activities of Daily Living (IADL), subjective health status, disability status diagnosed by a physician, activity limitation due to health conditions, and physician-diagnosed chronic diseases (hypertension, diabetes mellitus, cancer or malignancy, chronic obstructive pulmonary disease (COPD), liver disease, coronary heart disease, cerebrovascular disease, arthritis or rheumatism, gastrointestinal disorders, spinal disc disorders, mild cognitive impairment (MCI)). Other health-related variables included smoking status, alcohol consumption, vision and hearing-related limitations in daily activities, pain-related functional limitations, body mass index (BMI), and General Oral Health Assessment Index (GOHAI).

Employment-related variables included current economic activity and employment status. Income and asset-related factors included annual personal income, average monthly living expenses over the past year, and household net assets. Subjective expectations and quality of life variables included overall life satisfaction, satisfaction with relationships with children, satisfaction with financial status, satisfaction with health status, perceived social class, and expected life span (Table 1).

### 2.3. Data Preprocessing

The dataset was partitioned into a training set (70%, n = 706) and a test set (30%, n = 301) by stratified random sampling on depression risk before any preprocessing. Missing values were imputed separately within each set using the missForest package (version 1.5) in R (version 4.4.3; R Foundation for Statistical Computing, Vienna, Austria), with the outcome variable excluded from the imputation procedure. Imputing the two sets separately, rather than the combined dataset, prevented information from the test set from influencing the imputation of the training data. All subsequent preprocessing steps were based exclusively on the training set: normalization ranges and one-hot encoding levels were determined from the training data, and multicollinearity filtering and LASSO selection were performed using the training data alone. Model hyperparameters were also selected using only the training set, and the resulting models were then applied to the test set. SMOTE was restricted to the training data, while the test set retained its original class distribution. Apart from its separate imputation, the test set was not used to determine normalization ranges or one-hot encoding levels, perform multicollinearity filtering or feature selection, tune hyperparameters, or fit the models.

#### 2.3.1. Missing Data Imputation

Based on prior research [40], variables with more than 30% missing data were excluded to minimize bias. As a result, 46 variables were retained for analysis, including six categorical and eight continuous variables with less than 30% missingness. The categorical variables and their missing rates were medical insurance type (0.40%), disability status (10.43%), cerebrovascular disease diagnosis (0.79%), visual limitations (0.50%), pain-related functional limitations (20.75%), and perceived social class (0.10%). The continuous variables and their missing rates were personal annual income (2.48%), total financial support received (20.66%), monthly living expenses (1.59%), household net assets (4.17%), BMI (1.59%), satisfaction with child relationship (4.97%), number of siblings (21.75%), and expected life span (0.30%).

Missing values were imputed using the missForest algorithm, a non-parametric imputation algorithm based on random forests that can handle both continuous and categorical data while accounting for variable interactions [41]. After imputation, continuous variables were normalized using Min–Max scaling, and categorical variables were transformed using one-hot encoding so that no artificial ordinal relationship was imposed among categories; model complexity was subsequently controlled through LASSO-based feature selection and hyperparameter tuning.

#### 2.3.2. Feature Selection

Selecting meaningful predictors is crucial to enhancing both the predictive performance and interpretability of machine learning models. Removing irrelevant or weakly associated variables helps prevent overfitting and improves generalizability [42]. In this study, variables with high multicollinearity (|r_s_| > 0.7) were first excluded, and logistic LASSO regression was applied to the training dataset [40,43].

LASSO, which incorporates L1 regularization, shrinks the coefficients of non-essential variables to zero, thereby facilitating variable selection and reducing model complexity while maintaining predictive accuracy [44]. Using 10-fold cross-validation, the lambda value corresponding to the minimum cross-validation deviance (lambda.min) was selected (Supplementary Figure S1). At this lambda value, 23 predictor features were retained. The coefficient profiles across values of lambda are presented in Supplementary Figure S2, illustrating how the coefficients changed with increasing regularization.

#### 2.3.3. Addressing Class Imbalance with SMOTE

The dataset was partitioned into a training set (70%, n = 706) and a test set (30%, n = 301) by stratified random sampling prior to model development. To reduce class imbalance, which can bias model performance toward the majority class, SMOTE was applied only to the training data [45]. During hyperparameter tuning, SMOTE was applied separately within each training fold and was not applied to the corresponding validation fold. Before applying SMOTE, the proportions of individuals without and with depression risk were 68.84% and 31.16%, respectively. After SMOTE, these proportions were 52.48% and 47.52%, respectively (Table 2).

### 2.4. Machine Learning Methods and Performance Metrics

#### 2.4.1. Machine Learning Algorithms

Six machine learning algorithms were implemented to construct predictive models: Logistic Regression, Naive Bayes, Random Forest, Support Vector Machine (SVM), eXtreme Gradient Boosting (XGBoost), and Light Gradient Boosting Machine (LightGBM). Hyperparameter optimization, where applicable, was performed using grid search combined with 5-fold cross-validation.

A brief description of each algorithm is provided below:

1. Logistic Regression

A traditional statistical method based on the sigmoid function, logistic regression estimates the probability of a binary event (0 or 1). It remains one of the fundamental tools for risk prediction due to its simplicity and interpretability [46,47].

2. Naive Bayes

Naive Bayes is a simple probabilistic classifier that applies Bayes’ theorem with strong (naive) independence assumptions among features. It is particularly effective for high-dimensional data due to its computational efficiency [48,49].

3. Random Forest

Random Forest is an ensemble learning method that constructs multiple decision trees using bootstrap sampling. It offers high stability and prediction accuracy and is widely adopted in classification tasks [50].

4. SVM (Support Vector Machine)

SVM is a supervised learning algorithm that maps input data into high-dimensional space to maximize the margin between classes. It performs well in high-dimensional settings and is less prone to overfitting [51].

5. XGBoost (eXtreme Gradient Boosting)

XGBoost is an optimized implementation of gradient boosting that sequentially constructs trees to minimize a regularized objective function, offering computational efficiency and strong predictive performance [52].

6. LightGBM (Light Gradient Boosting Machine)

LightGBM is designed to improve the training efficiency of gradient boosting by adopting a leaf-wise rather than a level-wise tree growth strategy. In this approach, the leaf producing the greatest reduction in loss is split at each step [53].

#### 2.4.2. Hyperparameter Settings

Logistic Regression and Naive Bayes were fitted using prespecified default settings without hyperparameter tuning. The remaining four algorithms were tuned by grid search with 5-fold cross-validation on the training set: mtry for Random Forest, cost and gamma for the Support Vector Machine, maximum depth and learning rate for XGBoost, and learning rate, number of leaves, and maximum depth for LightGBM (27 combinations). Tuning was carried out separately for the training data with and without SMOTE, so that for the support vector machine and for LightGBM, the selected values differ between the two. The final settings for all six algorithms are given in Table 3.

#### 2.4.3. Explainable Artificial Intelligence (XAI) Approach

To enhance the interpretability of the depression risk prediction model for older adults living alone, the SHAP (SHapley Additive exPlanations) framework was employed. SHAP is based on Shapley values from cooperative game theory and provides fair attribution scores for individual features. It is widely recognized for satisfying both consistency and local accuracy in model interpretation [54]. Through SHAP analysis, key features contributing to the model predictions were identified and visually presented, thereby improving the model’s transparency and facilitating interpretation of its predictions.

To examine whether the feature-importance rankings depended on model choice, an exploratory comparison was additionally conducted across the four algorithms with the highest AUCs in the prespecified test set, namely LightGBM, Random Forest, Logistic Regression, and XGBoost. All four models used the same 23 predictors selected in the primary analysis and were fitted with SMOTE applied only to the training data. Importance was measured using mean absolute SHAP values for LightGBM and XGBoost, permutation importance for Random Forest, and absolute z values for Logistic Regression. Because the importance measures differed across algorithms, only their within-model ranks, rather than their absolute magnitudes, were compared, and agreement with the LightGBM ranking was summarized using Spearman rank correlations across all 23 predictors (Supplementary Table S3).

### 2.5. Statistical Analysis

All statistical analyses were conducted using R version 4.4.3 (R Foundation for Statistical Computing, Vienna, Austria) and Python version 3.10.16 (Python Software Foundation, Wilmington, DE, USA). To examine differences in participant characteristics based on depression risk, categorical variables were summarized using frequencies (n) and percentages (%) and analyzed using the chi-square test. A *p*-value of less than 0.05 was considered statistically significant. For continuous variables, normality was assessed using the Shapiro–Wilk test. None of the continuous variables was normally distributed. All continuous variables were therefore summarized as medians and interquartile ranges (IQRs) and compared using the Wilcoxon rank-sum test.

Model performance was summarized using the area under the receiver operating characteristic curve (AUC), the area under the precision–recall curve (PR-AUC), and the Brier score. Accuracy, sensitivity, specificity, positive predictive value, negative predictive value, and the F1 score were calculated at a classification threshold of 0.5. For the selected model, corresponding 95% CIs were obtained from 2000 bootstrap resamples of the test set. Accuracy alone is insufficient for evaluating model performance in this imbalanced classification setting, in which 31% of the sample was at risk of depression. Performance was therefore evaluated using discrimination and threshold-based metrics together. To assess the stability of performance beyond a single partition, stratified 5-fold cross-validation was repeated 20 times (100 folds). Within each fold, missing values were imputed separately in the training and validation portions. All subsequent preprocessing parameters were derived from the training portion and applied to the validation portion, and SMOTE was restricted to the training portion (Supplementary Methods S1). As a sensitivity analysis, the algorithms were refitted under the same scheme without LASSO pre-selection. The algorithm that yielded the highest AUC in the prespecified test split, with PR-AUC as a secondary criterion, was taken forward for the explainability analysis. Accuracy was not used for this purpose. This selection criterion was used to identify the model for the explainability analysis and was not intended to establish superiority over the other algorithms.

## 3. Results

### 3.1. General Characteristics by Depression Risk Status

Table 4 presents the differences in general characteristics of older adults living alone according to depression risk. Among demographic variables, statistically significant differences between the depression-risk groups were observed for age and frequency of contact with close acquaintances (*p* < 0.05). However, gender, education level, marital status, religion, type of residence, residential region, and type of medical coverage did not differ significantly between groups (*p* > 0.05).

Among family-related variables, neither the number of surviving siblings nor the total amount of financial support received from family members showed statistical significance (*p* > 0.05). With respect to health status, significant differences were found in ADL, IADL, BMI, subjective health status, activity limitation due to health, diagnoses of diabetes mellitus, coronary heart disease, cerebrovascular disease, regular exercise, hearing-related limitations in daily activities, pain-related functional limitations, and GOHAI (*p* < 0.05). Other health-related variables including physician-diagnosed disability, hypertension, cancer or malignancy, COPD, liver disease, arthritis or rheumatism, gastrointestinal disorders, spinal disc disorders, mild cognitive impairment, smoking, alcohol consumption, and vision-related limitations in daily activities were not significantly different between groups (*p* > 0.05).

Among employment-related variables, both current economic activity and current employment status differed significantly between groups (*p* < 0.05). Regarding income, consumption, and assets, personal annual income and average monthly living expenses were significantly different between groups (*p* < 0.05), whereas household net assets were not (*p* > 0.05). For subjective expectations and quality of life, all variables—including satisfaction with health status, financial satisfaction, satisfaction with relationships with children, overall life satisfaction, perceived social class, and expected lifespan—showed statistically significant differences (*p* < 0.05).

### 3.2. Comparison of Predictive Performance Across Models

Six machine learning models—Logistic Regression, Naive Bayes, Random Forest, SVM, XGBoost, and LightGBM—were trained to predict depression risk among older adults living alone. The models were trained with and without SMOTE applied to the training data and were evaluated on the same unchanged test set. Performance was compared using accuracy, ROC-AUC, and PR-AUC (Table 5).

**Figure 2:**
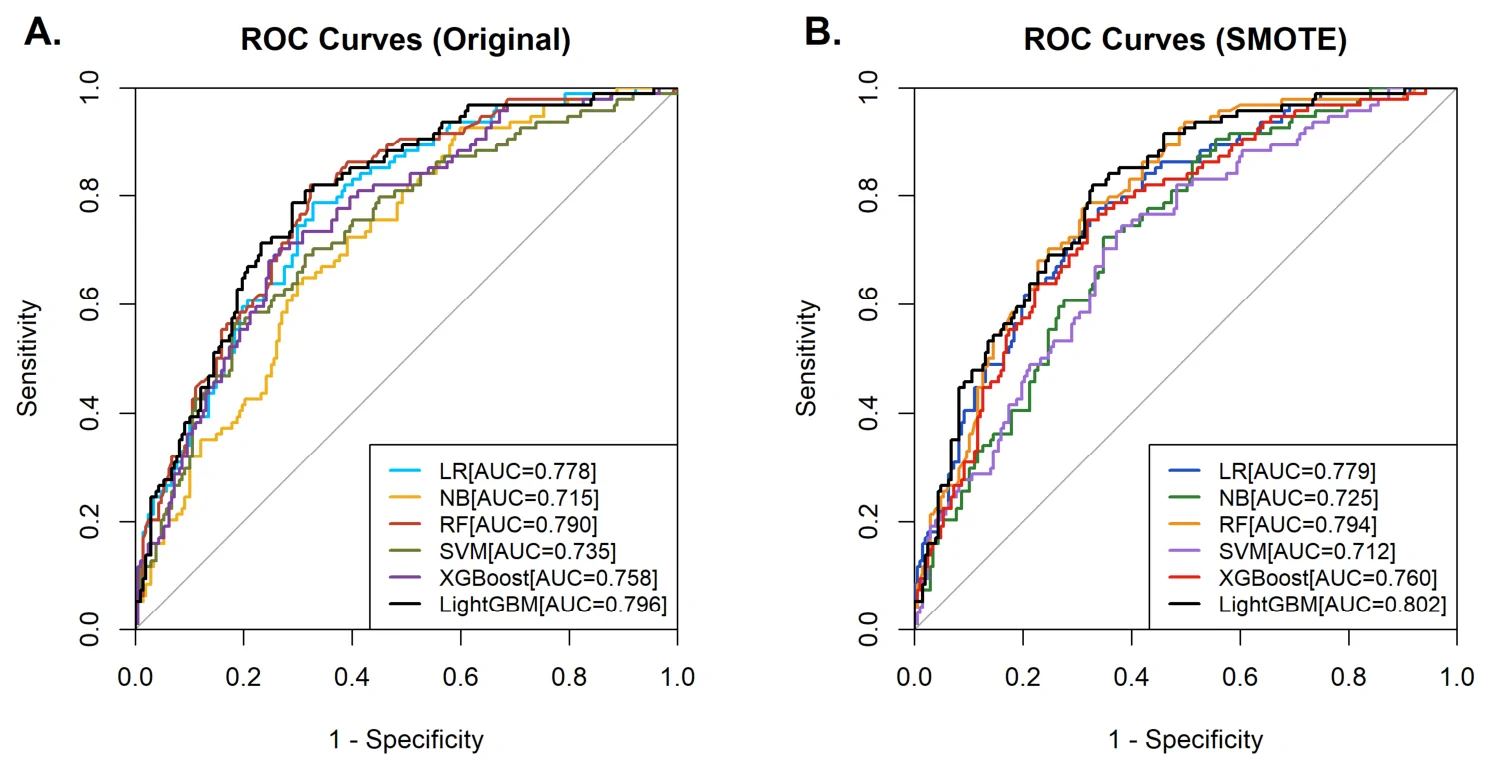
Comparison of the ROC Curves for Six Predictive Models. (**A**) without SMOTE and (**B**) with SMOTE applied to the training data. The gray diagonal line represents the line of no discrimination (AUC = 0.50), corresponding to the performance expected from random classification.

Among the models trained without SMOTE, LightGBM achieved the highest AUC (0.796), followed by Random Forest (0.790) and Logistic Regression (0.778), whereas Random Forest achieved the highest accuracy (0.741). When SMOTE was applied to the training data, ROC-AUC improved slightly for most algorithms, although it decreased for SVM (Figure 2). LightGBM again achieved the highest ROC-AUC (0.802), followed by Random Forest (0.794) and Logistic Regression (0.779). Random Forest achieved the highest accuracy (0.751), followed by LightGBM (0.741). The differences in discrimination among the three leading algorithms were small. The 95% CI for the AUC of the final SMOTE-based LightGBM model was 0.747–0.852.

The effect of SMOTE differed across algorithms and was small in terms of discrimination. For LightGBM, the AUC increased from 0.796 to 0.802, whereas for SVM it decreased from 0.735 to 0.712. SMOTE had a more pronounced effect on threshold-based performance. At the 0.5 threshold, the sensitivity of LightGBM increased from 0.277 to 0.585 and its F1 score increased from 0.388 to 0.585, whereas its specificity decreased from 0.932 to 0.812. The Brier score increased from 0.173 to 0.183, consistent with the overprediction of absolute risk shown in Supplementary Figure S3. Based on the selection criteria described in the Methods, the SMOTE-based LightGBM model was used for the subsequent explainability analysis. At the 0.5 threshold, this model identified 55 of the 94 participants at risk of depression and correctly classified 168 of the 207 participants who were not at risk. The complete set of performance measures for this model, each with a 95% confidence interval, is presented in Supplementary Table S2.

In repeated stratified cross-validation (100 folds), using the fold-specific preprocessing procedures described in Supplementary Methods S1 and fixed hyperparameter settings, the mean AUC was 0.776 (95% empirical interval, 0.720–0.832) for LightGBM, 0.783 for Random Forest, 0.770 for XGBoost, and 0.764 for Logistic Regression (Supplementary Table S1). The ordering of LightGBM and Random Forest was reversed relative to the single-split results, and the variation across folds exceeded the differences between algorithms. Thus, the present data did not provide clear evidence of differences in discrimination among the leading algorithms.

### 3.3. Identification of Key Depression Risk Factors

LightGBM yielded the highest AUC (0.802) and the highest area under the precision–recall curve (0.621) in the prespecified test set and was therefore examined using SHAP as an illustrative high-performing model. To assess whether the identified features depended strongly on this model choice, predictor-importance rankings were compared across the four algorithms with the highest test-set AUCs: LightGBM, Random Forest, Logistic Regression, and XGBoost. Rank agreement with the LightGBM ranking reported in Table 6 was high for the tree-based algorithms (Spearman rho across all 23 predictor features, 0.851 for XGBoost and 0.789 for Random Forest) and moderate for Logistic Regression (0.675), and oral health-related quality of life was ranked first by all four algorithms. Several of the leading features were therefore broadly consistent across algorithms, although their rankings varied (Supplementary Table S3).

**Figure 3:**
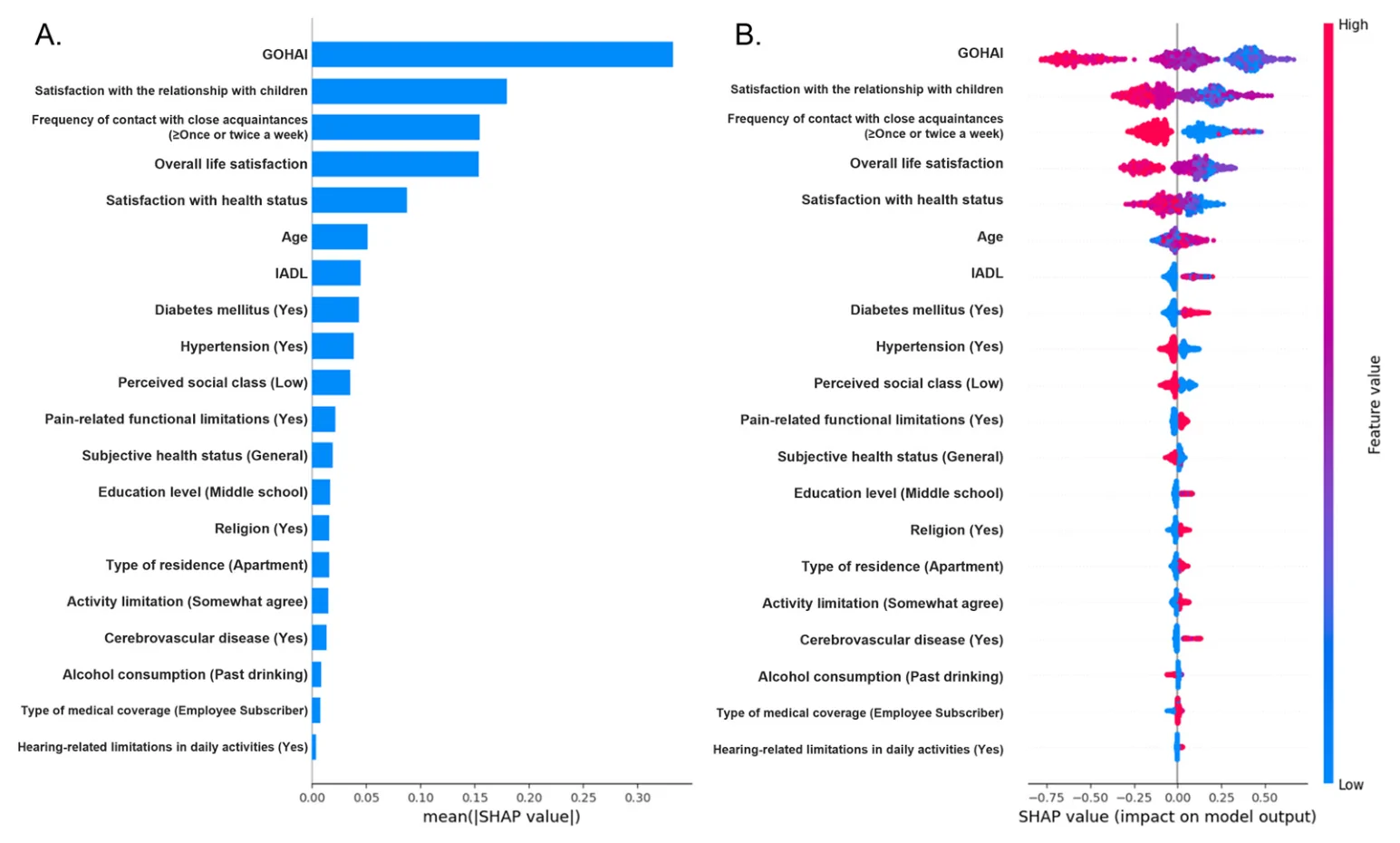
Feature importance analysis on the depression risk prediction model for older adults living alone based on SHAP values. (**A**) SHAP-based feature importance. (**B**) SHAP summary plot visualizing individual-level feature contributions to model predictions. Only the 20 highest-ranked features are displayed.

SHAP values were calculated for the selected LightGBM model to visualize important features associated with depression risk (Figure 3). Figure 3A shows that GOHAI, satisfaction with relationships with children, frequency of contact with close acquaintances, overall life satisfaction, satisfaction with health status, and age were the most influential features. IADL, diabetes mellitus, hypertension, and perceived social class also contributed to predictions, albeit to a lesser extent. Figure 3B illustrates the influence of feature values on depression risk predictions. Red indicates higher feature values, while blue indicates lower values. Features with positive SHAP values contribute to higher depression risk predictions, whereas those with negative values contribute to lower risk. GOHAI showed the largest mean absolute SHAP value and the widest SHAP distribution. Lower GOHAI scores were associated with higher predicted depression risk, whereas higher scores were associated with lower predicted risk. Similarly, lower scores for satisfaction with relationships with children, overall life satisfaction, and satisfaction with health status were associated with higher predicted depression risk. More frequent contact with close acquaintances was associated with lower predicted depression risk. Older age was also associated with higher predicted depression risk. A complete ranking of feature importance and SHAP values is presented in Table 6.

### 3.4. Sensitivity Analyses

Omitting LASSO pre-selection and refitting the algorithms using all candidate predictors remaining after multicollinearity filtering changed discrimination only marginally for the tree-based models (LightGBM 0.781 versus 0.776; Random Forest 0.785 versus 0.783; XGBoost 0.776 versus 0.770) and Logistic Regression (0.759 versus 0.764), while reducing it for SVM (0.699 versus 0.726) and Naive Bayes (0.666 versus 0.712). Omitting LASSO pre-selection therefore did not materially alter the mean AUC of the tree-based models or Logistic Regression, whereas SVM and Naive Bayes performed better with LASSO pre-selection in this repeated cross-validation analysis (Supplementary Table S4).

## 4. Discussion

This study developed machine learning models to predict depression risk among older adults living alone. Among the tested models, LightGBM showed the highest discrimination in the prespecified test set. However, its advantage over Random Forest and Logistic Regression was small, and Random Forest showed a slightly higher mean AUC than LightGBM in repeated cross-validation. Therefore, the leading models should be regarded as performing comparably in this sample. SHAP analysis further identified GOHAI, satisfaction with relationships with children, frequency of contact with close acquaintances, overall life satisfaction, satisfaction with health status, and age as the most influential features. The broadly similar importance rankings across the four highest-performing algorithms suggest that several leading features were not specific to LightGBM, although the degree of agreement varied across models and importance measures.

Among these, GOHAI emerged as the most important predictor. Higher GOHAI scores, indicating better oral health-related quality of life, were associated with lower predicted depression risk. This finding aligns with previous studies that employed CES-D-10 and confirmed the association between oral health and depression [55,56,57]. Similar findings were also reported using OHIP-14 and subjective oral health status [33,58].

In the present model, higher overall life satisfaction, satisfaction with relationships with children, and satisfaction with health status were associated with lower predicted depression risk, consistent with previous longitudinal findings [59]. Additionally, older adults with positive relationships with their children exhibited lower depressive symptoms, as previously reported [34]. Satisfaction with health status was also shown to be a key factor for emotional stability, with lower satisfaction associated with higher depression risk [60,61,62].

Frequent contact with close acquaintances was associated with lower predicted depression risk, consistent with findings that social engagement mitigates isolation and improves mental well-being [63,64]. Finally, older age was associated with higher predicted depression risk. Prior evidence indicates that the likelihood of depressive symptoms among older adults living alone increases with advancing age, being highest among the oldest-old, consistent with the present findings [65]. Furthermore, longitudinal evidence suggests that increased social isolation among older adults is linked not only to greater depressive symptoms but also to higher risks of disability, dementia, and mortality [66].

This study not only developed a machine learning model for depression risk prediction in older adults living alone but also visualized the influence of key factors using explainable AI. Unlike prior studies focused on linear associations, this study applied machine learning methods to explore potentially non-linear and complex predictive patterns. The focus on older adults living alone, a group more vulnerable than the general elderly population, further strengthens the study’s contribution. SHAP visualization enabled intuitive interpretation of individual feature contributions.

Limitations include the use of the self-reported CES-D-10, which may introduce response bias. Although a cutoff of 10 or above is commonly used to identify elevated depressive symptoms, it does not establish a clinician-assigned diagnosis and its classification performance may vary across populations. In addition, the analysis was based solely on cross-sectional data from 2022, limiting causal inference. Furthermore, no independent external cohort was available, so validation rested on a single internal partition supplemented by repeated cross-validation. The fold-to-fold spread observed in repeated cross-validation shows that performance in a sample of this size was estimated with appreciable uncertainty. External validation in a separate cohort and temporal validation using later waves of the KLoSA remain necessary before the model can be used in practice. It should also be noted that every predictor was based on a self-reported survey item rather than a clinical examination or laboratory measurement. Taken together with the moderate sensitivity obtained at the 0.5 threshold and the variation in performance across repeated cross-validation, these findings indicate that survey data of this kind should not be used on their own for individual-level clinical classification of depression risk. The model is therefore presented as an aid to screening rather than as a diagnostic instrument. The model is therefore presented as an aid to screening rather than as a diagnostic instrument. Although the exploratory cross-algorithm comparison showed broadly similar feature-importance rankings, the stability of these rankings across resampled datasets and under a common importance framework requires further evaluation. Future studies should compare predictor-selection frequencies and feature-importance rankings across repeated cross-validation or bootstrap samples and confirm their reproducibility in an independent cohort. The set-specific MissForest imputation used here avoided any flow of information from the test set to the training data, but future external validation should evaluate preprocessing procedures that can be applied prospectively to individual new observations.

Future directions include the use of longitudinal designs to track changes in depression risk over time, incorporation of clinical diagnostic data, and comparative studies on depression risk pre- and post-COVID-19. Future studies should validate the model against clinician-assigned diagnoses of depression, compare feature-selection strategies in an independent sample, and conduct stratified analyses by gender, age group, socioeconomic status, and urban–rural residence using adequate subgroup sizes.

## 5. Conclusions

This study developed an explainable machine learning model utilizing the 9th wave of the KLoSA dataset to predict depression risk among older adults living alone by integrating demographic, health, economic, social, and psychological variables. Among the tested algorithms, LightGBM achieved the highest AUC in the prespecified test set, with discrimination comparable to that of Random Forest and Logistic Regression, while SHAP analysis identified GOHAI, satisfaction with relationships with children, frequency of contact with close acquaintances, overall life satisfaction, satisfaction with health status, and age as the features contributing most to the model predictions. Notably, higher GOHAI scores were associated with lower predicted depression risk, highlighting oral health-related quality of life as a prominent feature in the model predictions. Furthermore, life satisfaction, social engagement, and positive family relationships were associated with lower predicted depression risk, whereas advanced age was associated with higher predicted risk. By employing explainable artificial intelligence to visualize feature contributions, this study extended previous findings beyond linear models and provided additional insight into potentially complex, non-linear patterns contributing to depression risk predictions among older adults living alone. These findings suggest that multidimensional and interpretable AI-based approaches may inform future screening strategies and further clinical assessment in this high-risk population.

## Figures and Tables

**Table 1:** Predictor Variables in Older Adult Living Alone.

Predictor Variable (n)		
Demographic factors (9)	Gender	Male = 0, Female = 1
	Age, years	65–103
	Education level	Elementary school = 1, Middle school = 2, High school = 3, College = 4
	Marital status	Never married = 0, Ever married = 1
	Religion	No = 0, Yes = 1
	Type of medical coverage	Employee subscriber = 1, Regional subscriber = 2, Medical aid (Type I/II) = 3
	Type of residence	House = 1, Apartment = 2
	Residential region	Urban (Dong) = 0, Rural area (Eup/Myeon) = 1
	Frequency of contact with close acquaintances	Once or twice a year = 1, Once or twice a month = 2, Once or twice a week = 3
Family Factors (2)	Number of surviving siblings	0–11
	Total amount of financial support	Continuous variable (Unit: 10,000 KRW)
Disease and health behavior factors (24)	Hypertension	No = 0, Yes = 1
	Diabetes mellitus	No = 0, Yes = 1
	Cancer or malignancy	No = 0, Yes = 1
	COPD	No = 0, Yes = 1
	Liver disease	No = 0, Yes = 1
	Coronary heart disease	No = 0, Yes = 1
	Cerebrovascular disease	No = 0, Yes = 1
	Arthritis or rheumatism	No = 0, Yes = 1
	Gastrointestinal disorders	No = 0, Yes = 1
	Spinal disc disorders	No = 0, Yes = 1
	Mild cognitive impairment (MCI)	No = 0, Yes = 1
	Disability status diagnosed	No = 0, Yes = 1
	Vision-related limitations in daily activities	No = 0, Yes = 1
	Hearing-related limitations in daily activities	No = 0, Yes = 1
	Pain-related functional limitations	No = 0, Yes = 1
	Subjective health status	Bad = 1, General = 2, Good = 3
	ADL	0–7
	IADL	0–10
	BMI	15–37
	Oral health status (GOHAI)	5–60
	Regular exercise	No = 0, Yes = 1
	Smoking status	Non-smoker = 1, Former smoker = 2, Current smoker = 3
	Alcohol consumption	Non-drinking = 1, Past drinking = 2, Current drinking = 3
	Activity limitation	Strongly agree = 1, Somewhat agree = 2, Somewhat disagree = 3, Strongly disagree = 4
Employment Factors (2)	Current economic activity status	No = 0, Yes = 1
	Current employment status	No = 0, Yes = 1
Economic factors (3)	Annual personal income	Continuous variable (Unit: 10,000 KRW)
	Average monthly living expenses over the past year	Continuous variable (Unit: 10,000 KRW)
	Household net assets	Continuous variable (Unit: 10,000 KRW)
Subjective expectations and quality of life factors (6)	Satisfaction with health status	0 (Not satisfied at all)–100 (Very satisfied)
	Satisfaction with financial status	0 (Not satisfied at all)–100 (Very satisfied)
	Satisfaction with relationships with children	0 (Not satisfied at all)–100 (Very satisfied)
	Overall life satisfaction	0 (Not satisfied at all)–100 (Very satisfied)
	Perceived social class	High = 1, Middle = 2, Low = 3
	Expected life span	0–100

**Table 2:** Number of samples before and after applying SMOTE to the training data.

Sample Type	Depression Risk		Total
	No	Yes	
Original	486 (68.84%)	220 (31.16%)	706 (100%)
SMOTE (oversampling)	486 (52.48%)	440 (47.52%)	926 (100%)

**Table 3:** Hyperparameter settings of the six algorithms.

Algorithm	Tuning	Setting
Logistic regression	Not tuned	Default settings
Naive Bayes	Not tuned	Default settings
Random forest	Grid search (mtry), 5-fold CV	mtry = 4; ntree = 500; nodesize = 5
Support vector machine	Grid search (cost, gamma), 5-fold CV	RBF kernel; gamma = 0.1; cost = 10 with SMOTE, cost = 1 without SMOTE
XGBoost	Grid search (max_depth, eta), 5-fold CV	max_depth = 4; eta = 0.1; 100 rounds
LightGBM	Grid search over 27 combinations	learning rate = 0.01; num_leaves = 31; 100 rounds; feature_fraction = 0.8; bagging_fraction = 0.8; bagging_freq = 5; max_depth = 5 with SMOTE, unlimited without SMOTE

**Table 4:** Characteristics of Older Adults Living Alone Categorized by Depression Risk Status.

Variable	Depression Risk			Missing (n, %)	*p*-Value
	Overall (N = 1007)	Yes (N = 314)	No (N = 693)		
**Gender**					0.573
Male	154 (15.29%)	51 (33.1%)	103 (66.9%)		
Female	853 (84.71%)	263 (30.8%)	590 (69.2%)		
**Age, years**	80.0 [73.0–85.0]	81.5 [75.0–86.0]	79.0 [73.0–84.0]		<0.001
**Education level**					0.430
Elementary school	673 (66.83%)	213 (67.8%)	460 (66.4%)		
Middle school	159 (15.78%)	54 (34.0%)	105 (66.0%)		
High school	141 (14%)	36 (25.5%)	105 (74.5%)		
College	34 (3.38%)	11 (32.4%)	23 (67.6%)		
**Marital status**					0.274
Never married	16 (1.59%)	7 (43.7%)	9 (56.3%)		
Ever married	991 (98.41%)	307 (31.0%)	684 (69.0%)		
**Religion**					0.544
No	634 (62.94%)	202 (31.9%)	432 (68.1%)		
Yes	373 (37.06%)	112 (30.0%)	261 (70.0%)		
**Type of medical coverage**				4 (0.40%)	0.256
Regional Subscriber	196 (19.54%)	61 (31.1%)	135 (68.9%)		
Employee Subscriber	696 (69.39%)	209 (30.0%)	487 (70.0%)		
Medical Aid	111 (11.07%)	42 (37.8%)	69 (62.2%)		
**Type of residence**					0.554
House	642 (63.75%)	196 (30.5%)	446 (69.5%)		
Apartment	365 (36.25%)	118 (32.3%)	247 (67.7%)		
**Residential region**					0.052
Urban	682 (67.73%)	226 (33.2%)	456 (66.8%)		
Rural area	325 (32.27%)	88 (27.1%)	237 (72.9%)		
**Frequency of contact with close acquaintances**					<0.001
1–2 times/year	147 (14.60%)	72 (48.98%)	75 (51.02%)		
1–2 times/month	219 (21.75%)	92 (42.0%)	127 (58.0%)		
≥1–2 times/week	641 (63.65%)	150 (23.41%)	491 (76.59%)		
**Number of surviving siblings**	3.0 [2.0–4.0]	3.0 [2.0–4.0]	3.0 [2.0–4.0]	219 (21.75%)	0.349
**Total amount of financial support (10,000 KRW)**	200.0 [100–402.5]	227.5 [86.3–450.0]	200.0 [100.0–390.0]	208 (20.66%)	0.890
**Hypertension**					0.346
No	371 (36.84%)	109 (29.4%)	262 (70.6%)		
Yes	636 (63.16%)	205 (32.2%)	431 (67.8%)		
**Diabetes mellitus**					<0.001
No	704 (69.91%)	195 (27.7%)	509 (72.3%)		
Yes	303 (30.09%)	119 (39.3%)	184 (60.7%)		
**Cancer or malignancy**					0.730
No	928 (92.15%)	288 (31.0%)	640 (69.0%)		
Yes	79 (7.85%)	26 (32.9%)	53 (67.1%)		
**COPD**					0.268
No	966 (95.93%)	298 (30.8%)	668 (69.2%)		
Yes	41 (4.07%)	16 (39.0%)	25 (61.0%)		
**Liver disease**					0.704
No	975 (96.83%)	305 (31.3%)	670 (68.7%)		
Yes	32 (3.17%)	9 (28.1%)	23 (71.9%)		
**Coronary heart disease**					<0.001
No	846 (84.00%)	242 (28.6%)	604 (71.4%)		
Yes	161 (16.00%)	72 (44.7%)	89 (55.3%)		
**Cerebrovascular disease**				8 (0.79%)	<0.001
No	920 (92.09%)	273 (29.7%)	647 (70.3%)		
Yes	79 (7.91%)	38 (48.1%)	41 (51.9%)		
**Arthritis or rheumatism**					0.060
No	519 (51.54%)	148 (28.5%)	371 (71.5%)		
Yes	488 (48.46%)	166 (34.0%)	322 (66.0%)		
**Gastrointestinal disorders**					0.513
No	974 (96.72%)	302 (31.0%)	672 (69.0%)		
Yes	33 (3.28%)	12 (36.4%)	21 (63.6%)		
**Spinal disc disorders**					0.446
No	966 (95.93%)	299 (30.9%)	667 (69.1%)		
Yes	41 (4.07%)	15 (36.6%)	26 (63.4%)		
**Mild cognitive impairment (MCI)**					0.076
No	994 (98.71%)	307 (30.9%)	687 (69.1%)		
Yes	13 (1.29%)	7 (53.8%)	6 (46.2%)		
**Disability status diagnosed**				105 (10.43%)	0.118
No	895 (99.22%)	268 (29.9%)	627 (70.1%)		
Yes	7 (0.78%)	4 (57.1%)	3 (42.9%)		
**Vision-related limitations in daily activities**				5 (0.50%)	0.606
No	956 (95.41%)	298 (31.1%)	658 (68.9%)		
Yes	46 (4.59%)	16 (34.8%)	30 (65.2%)		
**Hearing-related limitations in daily activities**					<0.001
No	928 (92.15%)	275 (29.6%)	653 (70.4%)		
Yes	79 (7.85%)	39 (49.4%)	40 (50.6%)		
**Pain-related functional limitations**				209 (20.75%)	<0.001
No	451 (56.52%)	123 (27.3%)	328 (72.7%)		
Yes	347 (43.48%)	142 (40.9%)	205 (59.1%)		
**Subjective health status**					<0.001
Bad	377 (37.44%)	165 (43.8%)	212 (56.2%)		
General	443 (43.99%)	111 (25.1%)	332 (74.9%)		
Good	187 (18.57%)	38 (20.3%)	149 (79.7%)		
**ADL**	0.0 [0.0–0.0]	0.0 [0.0–0.0]	0.0 [0.0–0.0]		0.002
**IADL**	0.0 [0.0–0.0]	0.0 [0.0–1.0]	0.0 [0.0–0.0]		<0.001
**BMI** (kg/m^2^)	23.1 [21.4–25.3]	22.7 [20.9–25.2]	23.4 [21.5–25.4]	16 (1.59%)	0.045
**GOHAI**	37.0 [33.0–42.5]	33.0 [29.0–37.0]	39.0 [35.0–44.0]		<0.001
**Regular exercise**					<0.001
No	627 (62.27%)	222 (35.4%)	405 (64.6%)		
Yes	380 (37.73%)	92 (24.2%)	288 (75.8%)		
**Smoking status**					0.091
Non-smoker	862 (85.59%)	261 (30.3%)	601 (69.7%)		
Former smoker	110 (10.93%)	44 (40.0%)	66 (60.0%)		
Current smoker	35 (3.48%)	9 (25.7%)	26 (74.3%)		
**Alcohol consumption**					0.812
Non-drinking	651 (64.67%)	206 (31.6%)	445 (68.4%)		
Past drinking	224 (22.25%)	70 (31.3%)	154 (68.8%)		
Current drinking	132 (13.11%)	38 (28.8%)	94 (71.2%)		
**Activity limitation**					<0.001
Strongly agree	98 (9.73%)	58 (59.2%)	40 (40.8%)		
Somewhat agree	372 (36.94%)	132 (35.5%)	240 (64.5%)		
Somewhat disagree	440 (43.69%)	116 (26.4%)	324 (73.6%)		
Strongly disagree	97 (9.63%)	8 (8.2%)	89 (91.8%)		
**Current economic activity status**					0.003
No	843 (83.70%)	279 (33.1%)	564 (66.9%)		
Yes	164 (16.30%)	35 (21.3%)	129 (78.7%)		
**Current employment status**					0.003
No	844 (83.80%)	279 (33.1%)	565 (66.9%)		
Yes	163 (16.20%)	35 (21.5%)	128 (78.5%)		
**Annual personal income (10,000 KRW)**	870 [600.0–1426.0]	788.0 [580.0–1230.0]	920.0 [620.0–1470.0]	25 (2.48%)	0.006
**Average monthly living expenses over the past year (10,000 KRW)**	80.0 [60.0–100.0]	70.0 [60.0–100.0]	80.0 [60.0–100.0]	16 (1.59%)	0.035
**Household net assets (10,000 KRW)**	13,150.0 [5000.0–25,000.0]	13,000.0 [4300.0–25,000.0]	13,750.0 [5000.0–25,000.0]	42 (4.17%)	0.679
**Satisfaction with health status**	60.0 [40.0–70.0]	50.0 [30.0–60.0]	60.0 [50.0–70.0]		<0.001
**Satisfaction with financial status**	50.0 [40.0–70.0]	50.0 [30.0–60.0]	60.0 [50.0–70.0]		<0.001
**Satisfaction with relationships with children**	70.0 [60.0–80.0]	60.0 [50.0–70.0]	70.0 [60.0–80.0]	50 (4.97%)	<0.001
**Overall life satisfaction**	60.0 [50.0–70.0]	50.0 [40.0–67.5]	60.0 [50.0–70.0]		<0.001
**Perceived social class**				1 (0.10%)	<0.001
High	21 (2.09%)	12 (57.1%)	9 (42.9%)		
Middle	394 (39.17%)	100 (25.4%)	294 (74.6%)		
Low	591 (58.74%)	201 (34.0%)	390 (66.0%)		
**Expected life span**	50.0 [20.0–60.0]	40.0 [20.0–60.0]	50.0 [30.0–60.0]	3 (0.30%)	<0.001

**Table 5:** Comparison of Model Performance on the Test Set.

Algorithm	Original			SMOTE		
	Accuracy	ROC-AUC	PR-AUC	Accuracy	ROC-AUC	PR-AUC
Logistic regression	0.728	0.778	0.608	0.718	0.779	0.619
Naive Bayes	0.664	0.715	0.516	0.684	0.725	0.524
Random Forest	0.741	0.790	0.607	0.751	0.794	0.615
SVM	0.714	0.735	0.562	0.684	0.712	0.516
XGBoost	0.724	0.758	0.575	0.728	0.760	0.579
LightGBM	0.728	0.796	0.620	0.741	0.802	0.621

**Table 6:** Ranking of Feature Importance and SHAP Values.

Ranking	Feature	Mean (|SHAP|)
1	GOHAI	0.333
2	Satisfaction with the relationship with children	0.180
3	Frequency of contact with close acquaintances (≥Once or twice a week)	0.155
4	Overall life satisfaction	0.154
5	Satisfaction with health status	0.088
6	Age	0.051
7	IADL	0.045
8	Diabetes Mellitus (Yes)	0.043
9	Hypertension (Yes)	0.038
10	Perceived social class (low)	0.035
11	pain-related functional limitations (Yes)	0.021
12	Subjective health status (General)	0.019
13	Education level (Middle school)	0.017
14	Religion (Yes)	0.016
15	Type of residence (Apartment)	0.016
16	Activity limitation (Somewhat agree)	0.015
17	Cerebrovascular disease (Yes)	0.013
18	Alcohol consumption (Past drinking)	0.009
19	Type of medical coverage (Employee Subscriber)	0.008
20	Hearing-related limitations in daily activities (Yes)	0.004
21	Coronary heart disease (Yes)	0.003
22	Activity limitation (Strongly agree)	0.002
23	Cancer or malignancy (Yes)	0.001

## Data Availability

The data analyzed in this study were obtained from the Korean Longitudinal Study of Aging (KLoSA), administered by the Korea Employment Information Service (KEIS). The KLoSA data are available to researchers through the KEIS Employment Survey website (https://survey.keis.or.kr/ (accessed on 5 July 2025)) following the required data-use application procedure. As the original dataset is provided and administered by KEIS, the authors do not redistribute the original data directly. Researchers wishing to access the original data should obtain them directly from KEIS through the designated application process. Further information regarding the data used and analyzed in the present study is available from the corresponding author upon reasonable request.

## Supplementary Materials

The following supporting information can be downloaded at https://www.mdpi.com/article/10.3390/healthcare14183088/s1. Methods S1: Internal validation and stability assessment of the depression risk prediction models; Figure S1: Cross-validation deviance according to the LASSO regularization parameter; Figure S2: LASSO coefficient profiles across values of the regularization parameter; Figure S3: Calibration of the LightGBM models in the test set; Table S1: Model performance in repeated stratified cross-validation; Table S2: Full performance of the selected LightGBM model in the test set; Table S3: Predictor-importance ranks across four algorithms; Table S4: AUC with and without LASSO pre-selection.
