# Supplementary material for: Explainable AI for Digital Health: Predicting Depression Risk in Older Adults Living Alone Using Machine Learning
Source: Healthcare (Basel). 2026 Sep 19;14(18):3088. doi: 10.3390/healthcare14183088 (PMC13606131; doi:10.3390/healthcare14183088)
Supplement: Supplementary file 1 [file healthcare-14-03088-s001.zip › healthcare-4540379-supplementary.pdf]

Supplementary Materials

Methods S1. Internal validation and stability assessment of the depression risk prediction models

Table S1. Model performance in repeated stratified cross-validation

Table S2. Full performance of the selected LightGBM model in the test set

Table S3. Predictor-importance ranks across four algorithms

Table S4. AUC with and without LASSO pre-selection

Figure S1. Cross-validation deviance according to the LASSO regularization parameter

Figure S2. LASSO coefficient profiles across values of the regularization parameter

Figure S3. Calibration of the LightGBM models in the test set

Methods S1. Internal validation and stability assessment of the depression risk prediction models

To quantify the extent to which the reported performance depends on a single 70/30 partition, stratified 5-fold cross-validation was repeated 20 times, yielding 100 training-validation folds. Within each iteration, missing values were imputed separately in the training and validation portions using MissForest, with the outcome excluded. All subsequent preprocessing steps—including Min-Max scaling ranges, one-hot encoding levels, removal of variables with $|r_s| > 0.7$, and logistic LASSO selection at lambda.min—were fitted using the training fold and applied to the validation fold, and SMOTE was applied only to the training fold. The six algorithms were fitted using the hyperparameter settings specified for the models with SMOTE in **Table 3** of the main text. These settings were selected in the primary analysis and held fixed across folds. This analysis therefore represents repeated cross-validation using fixed hyperparameter settings rather than fully nested cross-validation. Threshold-based measures were calculated at a threshold of 0.5, and **Table S1** reports the mean of each measure across the 100 folds. The same scheme was repeated with the LASSO step omitted (**Table S4**).

Table S1. Model performance in repeated stratified 5-fold cross-validation (20 repetitions, 100 folds).

Algorithm	AUC, mean (95% empirical interval)	Sensitivity	Specificity	F1 score	PR-AUC	Brier score
Logistic regression	0.764 (0.708-0.818)	0.638	0.745	0.579	0.586	0.194
Naive Bayes	0.712 (0.642-0.784)	0.583	0.740	0.540	0.530	0.250
Random forest	0.783 (0.728-0.838)	0.505	0.853	0.551	0.597	0.172
SVM	0.726 (0.670-0.793)	0.343	0.871	0.411	0.519	0.200
XGBoost	0.770 (0.719-0.828)	0.533	0.817	0.549	0.585	0.181
LightGBM	0.776 (0.720-0.832)	0.551	0.812	0.560	0.590	0.177

Values are means across the 100 validation folds. Sensitivity, specificity, and the F1 score were calculated at a classification threshold of 0.5. Values in parentheses are the 2.5th and 97.5th percentiles of the fold-level AUC distribution and should not be interpreted as confidence intervals for the mean performance because the folds are not mutually independent. These estimates are not directly comparable with those in Table 5, which are based on models fitted to a single 70% training set and evaluated on the corresponding 30% test set

Table S2. Full test-set performance of the selected SMOTE-based LightGBM model (n = 301), with 95% confidence intervals from 2,000 bootstrap resamples.

Measure	Estimate	95% CI
AUC	0.802	0.747-0.852
Accuracy	0.741	0.691-0.791
Sensitivity	0.585	0.481-0.685
Specificity	0.812	0.754-0.864
Positive predictive value	0.585	0.484-0.684
Negative predictive value	0.812	0.755-0.864
F1 score	0.585	0.494-0.664
Area under the precision-recall curve	0.621	0.516-0.724
Brier score	0.183	0.170-0.197

Threshold-based measures were calculated at a classification threshold of 0.5. At this threshold, the model yielded 55 true positives, 39 false positives, 39 false negatives, and 168 true negatives. Sensitivity and positive predictive value coincide, as do specificity and negative predictive value, because the number of false positives equals the number of false negatives.

Table S3. Predictor-importance ranks across four algorithms for the 23 predictor features selected in the primary analysis.

Predictor	LightGBM (SHAP rank)	XGBoost SHAP rank	RF (permutation rank)	LR (|z| rank)
Oral health-related quality of life (GOHAI)	1	1	1	1
Satisfaction with the relationship with children	2	4	3	3
Frequency of contact with close acquaintances (≥ once or twice a week)	3	3	4	2
Overall life satisfaction	4	2	5	7
Satisfaction with health status	5	6	2	15
Age	6	5	6	17
IADL	7	14	7	16
Diabetes mellitus (yes)	8	8	8	6
Hypertension (yes)	9	7	17	4
Perceived social class (low)	10	9	10	5
Pain-related functional limitations (yes)	11	16	12	18
Subjective health status (general)	12	18	15	14
Education level (middle school)	13	13	9	8
Religion (yes)	14	19	20	12
Type of residence (apartment)	15	11	22	13
Activity limitation (somewhat agree)	16	17	19	19
Cerebrovascular disease (yes)	17	12	16	9
Alcohol consumption (past drinking)	18	15	21	11
Type of medical coverage (employee subscriber)	19	10	18	10
Hearing-related limitations in daily activities (yes)	20	23	14	21
Coronary heart disease (yes)	21	22	13	20
Activity limitation (strongly agree)	22	21	11	22
Cancer or malignancy (yes)	23	20	23	23

The comparison included the four algorithms with the highest AUCs in the prespecified test set. All four models used the same 23 predictors, training-test partition, and SMOTE-based training condition as the primary analysis. Importance was measured using mean absolute SHAP values for LightGBM and XGBoost, permutation importance for Random Forest, and absolute z values for Logistic Regression. Because the importance measures differed across algorithms, only their within-model ranks, rather than their absolute magnitudes, were compared, and ranks were assigned across all 23 predictors within each model. The LightGBM column reproduces the ranking reported in Table 6 of the main text, and the predictors are listed in that order. Spearman rank correlations with the LightGBM ranking, calculated across all 23 predictors, were 0.851 for XGBoost, 0.789 for Random Forest, and 0.675 for Logistic Regression.

Table S4. AUC with and without LASSO pre-selection in repeated cross-validation.

Algorithm	With LASSO pre-selection	Without pre-selection	Difference
Random forest	0.783	0.785	+0.002
LightGBM	0.776	0.781	+0.005
XGBoost	0.770	0.776	+0.006
Logistic regression	0.764	0.759	−0.005
SVM	0.726	0.699	−0.027
Naive Bayes	0.712	0.666	−0.046

Values are mean AUCs across the 100 validation folds. Difference was calculated as the mean AUC without LASSO pre-selection minus the mean AUC with LASSO pre-selection.

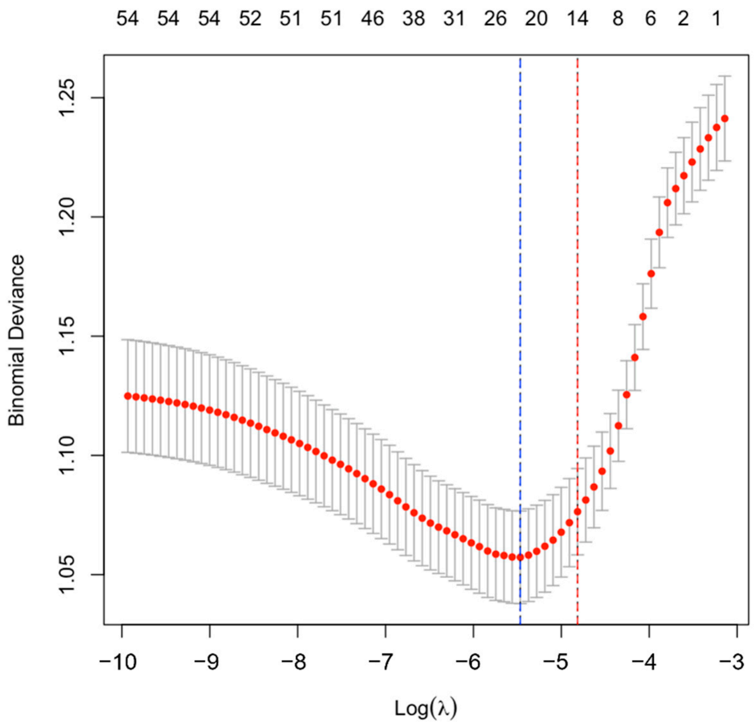

Figure S1. Cross-validation deviance according to the LASSO regularization parameter. The blue dashed line indicates the value of lambda giving the minimum deviance (lambda.min), and the red dashed line the largest lambda within one standard error of the minimum (lambda.1se).

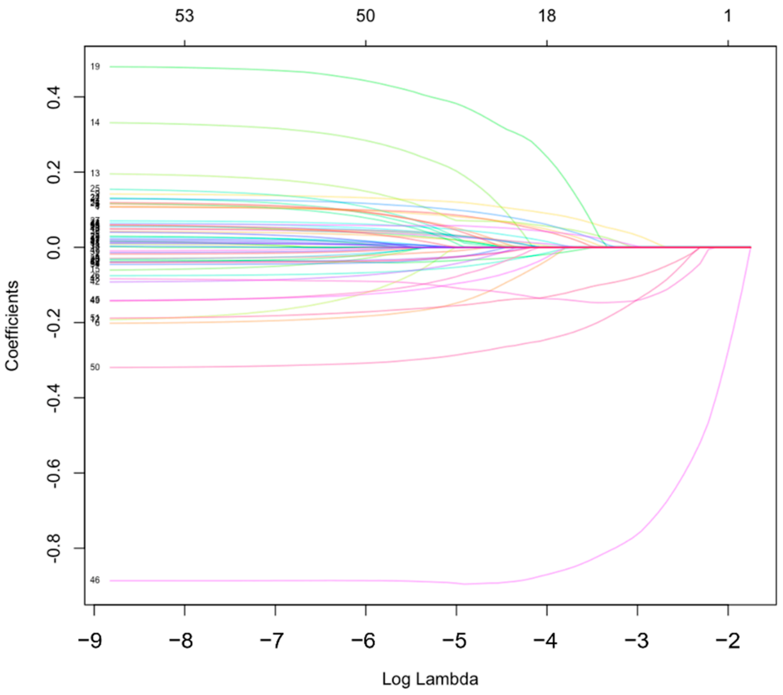

Figure S2. LASSO coefficient profiles across values of the regularization parameter.

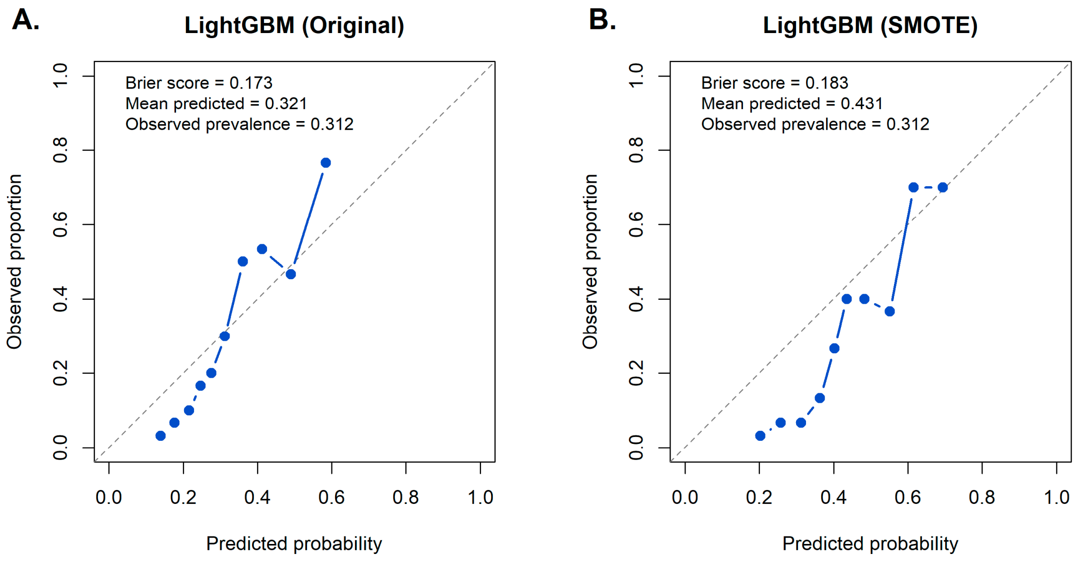

Figure S3. Calibration of the LightGBM models in the test set (n = 301), by deciles of predicted risk. (A) Model fitted without SMOTE; (B) model fitted with SMOTE. Points represent the observed proportions of participants at risk of depression within deciles of predicted risk. The dashed line indicates perfect calibration.
